# Occupational stress and musculoskeletal symptoms in a Tunisian footwear industry

**DOI:** 10.1192/j.eurpsy.2022.1603

**Published:** 2022-09-01

**Authors:** N. Rmadi, N. Kammoun, R. Masmoudi, N. Kotti, J. Masmoudi, K. Hammami, M.L. Masmoudi, M. Hajjaji

**Affiliations:** 1 HEDI CHAKER hospital, Department Of Occupational Medicine, SFAX, Tunisia; 2 occupational health and safety institute, Department Of Occupational Medicine, Tunis, Tunisia; 3 Hospital university of HEDI CHAKER, Psychiatry A Department, Sfax, Tunisia

**Keywords:** musculoskeletal disorder, occupational stress

## Abstract

**Introduction:**

A growing body of literature has documented that occupational stress is associated with increased risks of musculoskeletal injuries or symptoms.

**Objectives:**

The purpose of this study was to assess the effect of occupational stress on the occurrence of musculoskeletal symptoms among workers in a Tunisian footwear industry.

**Methods:**

Material and methods: This was an exhaustive and cross-sectional study including workers in a footwear industry. Musculoskeletal symptoms were assessed using a modified Nordic questionnaire. We used the Job Demand/Control model of Karasek to measure occupational stress. The Quick Exposure Check (QEC) method was used as an ergonomic risk assessment tool. Data were analysed using R software.

**Results:**

A total of 337 workers participated in the survey (the age range: 18-60 years). A total of 83.7 of workers reported musculoskeletal symptoms at one site or more. Elbows and upper back were the most symptomatic sites in respectively 84% and 65%. We noted job-strain and iso-strain situations in respectively 57% and 32%. In 78.1% of the workers studied, the QEC score was high and very high in 21.9%. Multivariable-adjusted logistic regression model showed that iso-strain situation was associated with the number of symptomatic sites (p= 0.0003, OR=1.34), having musculoskeletal symptoms in elbows (p= 0.03, OR=2.33) and upper back (p=0.009, OR=2.40), and the final QEC score (p= 0.018, OR= 1.04).

**Conclusions:**

Occupational stress constitutes a significant risk for this leather industry. It is associated with a higher prevalence of musculoskeletal symptoms in the workplace and with work-related biomechanical exposure.

**Disclosure:**

No significant relationships.

